# Risk factors limiting first service conception rate in dairy cows and their economic impact

**DOI:** 10.5713/ajas.18.0296

**Published:** 2018-09-13

**Authors:** Ill Hwa Kim, Jae Kwan Jeong

**Affiliations:** 1 Veterinary Medical Center and College of Veterinary Medicine, Chungbuk National University, Cheongju 28644, Korea

**Keywords:** Dairy Cow, First Service, Conception, Risk Factor, Economic Impact

## Abstract

**Objective:**

We determined the risk factors limiting first service conception (FSC) rate in dairy cows and their economic impact.

**Methods:**

Data were collected from 790 lactations regarding cow parity, peri- and postpartum disorders, body condition score (BCS), reproductive performance, and expenses associated with reproductive management (treatment, culling, and others). Initially, we identified the risk factors limiting FSC rate in dairy cows. Various biological and environmental factors, such as herd, cow parity, BCS at 1 month postpartum and first artificial insemination (AI), resumption of cyclicity within 1 month of calving, year, AI season, insemination at detected estrus or timed AI, peri- and postpartum disorders, and calving to first AI interval, were evaluated. Next, we evaluated the economic impact of the success or failure of FSC by comparing the expense associated with reproductive management until conception between cows that did or did not conceive at their first service.

**Results:**

Cows with BCS <3.0 had a lower probability of conceiving at first insemination (odds ratio [OR] = 0.64, p<0.05) than cows with BCS ≥3.0. Cows inseminated during summer were less likely to conceive (OR = 0.44, p<0.001) than cows inseminated during spring. Cows with peri- or postpartum disorders were less likely to conceive (OR = 0.55, p<0.001) than cows without disorders. Survival curves generated using MedCalc showed an 81 day extension in the mean interval between calving and conception in cows that failed to conceive over those that did conceive at first insemination. Cows failing conceive required additional expenditure on reproductive treatment ($55.40) and other management ($567.00) than cows that conceived at first insemination.

**Conclusion:**

Lower BCS, hot weather at first insemination, and peri- and postpartum disorders are risk factors limiting FSC, which result in an economic loss of $622.40 per dairy cow.

## INTRODUCTION

Reproductive performance is crucially important to maintain profitability in the dairy industry [1]. Conception at the first service after calving is key to optimal reproductive performance in dairy cows [2], although the percentage success of first service has been shown to range between 26.7% and 50.7% in previous studies [3,4]. A failure of first service conception (FSC) may lead to an increase in the number of days open, insemination numbers, reproductive treatment, feeding, culling, and replacement heifers [5,6]. Thus, the identification of factors that potentially limit the success of FSC, including biological and environmental conditions, might be useful to improve reproductive performance in dairy cows.

Diverse factors, e.g., body condition score (BCS) during transition or at service, heat stress, age or parity, milk yield, calving to first service interval, and peripartum disorders (dystocia, metritis, and retained placenta) have been interrogated for an effect on FSC [3,4,7]. Higher BCSs before calving [8] and at first service [3], and less BCS loss during the first month after calving [4] were associated with a higher FSC rate, although BCS at calving was not associated the FSC rate [4]. In a previous study, cow parities (four or greater), higher milk yield (>39 kg/d), and estimated genetic values were also associated with a higher risk of a low FSC rate in the west-central region of France [7]. One meta-analysis of data in 70 papers reported that clinical ketosis, dystocia, and retained placenta were associated with a slight decrease in the FSC rate (4% to 10%) and that metritis was associated with a moderate decrease (20%), whereas stillbirth, milk fever, abomasal displacement, and mastitis were not associated with FSC rate [9]. Conversely, another previous study showed that the birth of twins and stillbirth reduced the probability of FSC [2]. Still another study showed that short intervals from calving to first artificial insemination (AI), dystocia, autumn calvings, or a cow parity of five as well as postpartum disorders (metritis, hypocalcemia, and retained placenta) were associated with a higher risk of a low FSC rate in one area of northwest Spain [10]. Moreover, this study additionally reported that farms located in the central area of northwest Spain had a higher risk of low FSC rates than those located in the coastal area, possibly because of differences in management systems [10]. Likewise, the risk factors that have been identified as limiting FSC rate have varied among previous studies, probably due to different management and practice systems, characteristics of animals, and regional geography [9].

In Korea, continuous breeding for an increased milk yield and use of intensive production systems have dramatically increased milk production per cow, but decreased reproductive performance [11]. Moreover, since milk is very expensive in Korea and consequently the primary goal of farm management is to obtain a high milk yield, which further worsens reproductive outcomes. Thus, identification of risk factors limiting FSC in Korean dairy herds might provide valuable information that can be used to improve reproductive performance in dairy herds with high yields under intensive production systems. Therefore, the first objective of this study was to determine the biological (cow parity, BCS, resumption of postpartum cyclicity, timed AI, peri- and postpartum disorders, and calving to first service interval) and environmental (herd, year, and AI season) factors limiting FSC rate in Korean dairy herds.

Estimation of the economic effects of the success or failure of FSC might provide useful information for dairy farmers. However, such an assessment is difficult because of a number of factors, such as variation in the cost of the animal, reproductive efficiency, feed, and labor, in different countries. Therefore, the second objective of this study was to determine the economic impact of FSC rate in dairy cows in Korea.

## MATERIALS AND METHODS

### Animals

This study was conducted on two dairy farms (A and B), located in Chungcheong Province, Korea, during the period from 2011 to 2016. Each herd consisted of approximately 100 cows. Cows were maintained in loose housing systems, fed total mixed rations, and milked twice daily. The mixed rations were composed of brewers’ grain, alfalfa hay, cotton seed, beet pulp, corn silage, tall fescue, timothy hay, and mineral and vitamin additives. The mean milk yields for the farms were ~9,500 to 10,000 kg per cow per year. All experiments were performed according to the ethical guidelines based on the Institutional Animal Care and Use Committee of Chungbuk National University, Korea.

A total of 790 lactations (340 primiparous and 450 multiparous) in 426 cows were included in the study. All cows received biweekly reproductive health checks by veterinarians on the research team, which included an examination of their ovarian structures (follicle and corpus luteum [CL]) and uterus by transrectal palpation and ultrasonography (Sonovet 600 with 7.5 MHz linear-array transducer, Medison, Seoul, Korea).

### Study design

Initially, we identified the risk factors limiting FSC rate in dairy cows. Various biological and environmental factors, such as herd, cow parity, BCS at 1 month postpartum and first AI, resumption of cyclicity within 1 month of calving (determined by the existence of a CL using ultrasonography), year, AI season, insemination at detected estrus (IDE) or timed AI, peri- and postpartum disorders (dystocia, retained placenta, septicemic metritis, clinical endometritis, ketosis, milk fever, and abomasal displacement), and calving to first AI interval, were evaluated. Next, we evaluated the economic impact of the success or failure of FSC by comparing the expense associated with reproductive treatment (hormone treatment, semen, and palpation) and other reproductive management (replacement heifers, nutrition, calf price, and labor) until conception between cows that did or did not conceive at their first service.

### Case definitions

The definitions of peri- and postpartum health disorders that were used in the present study were similar to those described previously [12–14]. Calving difficulty was ranked according to the degree of assistance required (1 = no assistance, 2 = minor assistance, 3 = some force required, 4 = significant force required, and 5 = cesarean section). Cows with a calving score >2 were considered to have dystocia. Retained placenta was defined as the retention of the fetal membranes for longer than 24 h. Septicemic metritis was defined by the presence of fever (≥39.5°C) and a watery, fetid uterine discharge during the first 10 days postpartum. Ketosis was diagnosed by the following clinical signs within 4 weeks postpartum: anorexia, depression, and the odor of acetone on the breath. Milk fever was diagnosed by the presence of weakness and recumbency after calving. Abomasal displacement was diagnosed by the detection of a ‘ping’ sound during abdominal auscultation within 4 weeks postpartum. Clinical endometritis was diagnosed based on the presence of a visible mucopurulent vaginal discharge and/or rectal palpation of the enlarged uterus at 4 weeks postpartum.

### Evaluation of body condition score and reproductive management

BCS was evaluated at 1 month postpartum and when the first AI was performed. BCS was measured on a 5-point scale (with quarter-point divisions) using a visual technique [15].

Resumption of postpartum cyclicity was evaluated using ultrasonography and confirmed by detection of a CL within 4 weeks of calving. The voluntary waiting period from calving to first AI was 45 days. In addition to estrus detection, a herd reproductive management program was employed. Estrus synchronization was achieved by administration of prostaglandin (PG) F_2a_ (25 mg dinoprost, Lutalyse, Zoetis, Louvain-la-Neuve, Belgium) or by Ovsynch [16]. Ovsynch was performed with a combination of gonadotrophin releasing hormone (GnRH) (100 μg gonadorelin, Fertagyl, MSD Animal Health, Unterschleissheim, Germany) on day 0, PGF_2α_ on day 7, and GnRH on day 9. Ovsynch was performed with or without a controlled, internal drug-release device containing 1.9 g progesterone (EAZI-BREED CIDR, Zoetis, Auckland, New Zealand), which was inserted between days 0 and 7. Cows that exhibited estrus naturally or after synchronization using PGF_2α_ were inseminated according to the am-pm rule. Cows treated with Ovsynch were subjected to timed AI. Pregnancy diagnosis was performed 32 to 40 days after AI by transrectal palpation and ultrasonography. Reproductive performance data were collected for a minimum of 210 days postpartum, or until pregnancy or culling.

### Evaluation of the economic impact of first service conception

The expense associated with the success or failure of FSC includes the costs of reproductive treatment both for the cows that conceived and those that failed to conceive at their first AI, and the costs of other reproductive management procedures for cows that failed to conceive at their first AI, incurred because of a higher number of days open (mean interval between calving and conception) than for cows that did conceive at their first AI [17]. Thus, the cost of reproductive treatment was calculated firstly using the total costs of reproductive hormones (PGF_2a_, GnRH, and CIDR), semen, and palpation, until conception occurred. The cost of other reproductive management for cows that failed to conceive at their first AI comprised the costs of replacement heifers, nutrition, calf price, and labor, associated with the larger number of days open. Cows that were sold or which had died by 210 days postpartum were not included in the analyses.

### Statistical analyses

Results are expressed as the mean±standard error of the mean. For statistical analyses, calving season was categorized as spring (March to May), summer (June to August), autumn (September to November), or winter (December to February), while cow parity was categorized as 1, 2, 3, or ≥4. Statistical analyses were performed using the SAS program (version 9.4, SAS Inst., Cary, NC, USA).

The median and mean days from calving to conception for cows that did or did not conceive at first AI were determined by survival analysis using the Kaplan-Meier model and the LIFETEST procedure within the SAS software. A Cox proportional hazard model with the PHREG procedure was used to compare the hazards of conception by 210 days postpartum between cows that did or did not conceive at first AI. This model included BCS at 1 month postpartum (≥2.75 vs <2.75), IDE or timed AI, peri- and postpartum disorders (dystocia, retained placenta, septicemic metritis, clinical endometritis, ketosis, milk fever, and abomasal displacement), calving to first AI interval (<80 vs ≥80 days), cows that did or did not conceive at first AI, and the interactions between these variables. Cow was included in the model as a random effect. A survival plot was generated using the survival option within MedCalc software (11.1, MedCalc Software, Mariakerke, Belgium).

The risk factors for FSC were analyzed by logistic regression using the LOGISTIC procedure. First, we determined the relationships between FSC and variables (farm, cow parity [1, 2, 3, or ≥4], BCS at 1 month postpartum [≥2.75 vs <2.75] and first AI [≥3.0 vs <3.0], detection of a CL within 1 month of calving, year, AI season, IDE or timed AI, peri- and postpartum disorders [dystocia, retained placenta, septicemic metritis, clinical endometritis, ketosis, milk fever, and abomasal displacement], and calving to first AI interval) by performing univariate analysis. Thereafter, the risk factors limiting FSC were analyzed using a multiple logistic regression model. This model included BCS at first AI, AI season, and peri- and postpartum disorders, and the interactions between these variables. Cow was included in the model as a random effect. Backward stepwise regression was used in the multiple regression model, and elimination was performed based on the Wald statistic criterion when p>0.11. Odds ratios (ORs) and 95% confidence intervals (CIs) were determined by logistic regression. Results are presented as percentages and ORs with their respective 95% CIs. A p-value <0.05 was considered statistically significant.

## RESULTS

### Reproductive performance in the study herds

The mean intervals from calving to first insemination or conception, FSC rate, and insemination number per conception in the study herds were 91.2 days, 141.3 days, 42.3%, and 2.1, respectively. The culling rate owing to infertility in cows that did not conceive at their first AI was 15.8% (72/456), whereas no cows were culled because of infertility if they did conceive at their first AI (0/334).

**Figure 1:**
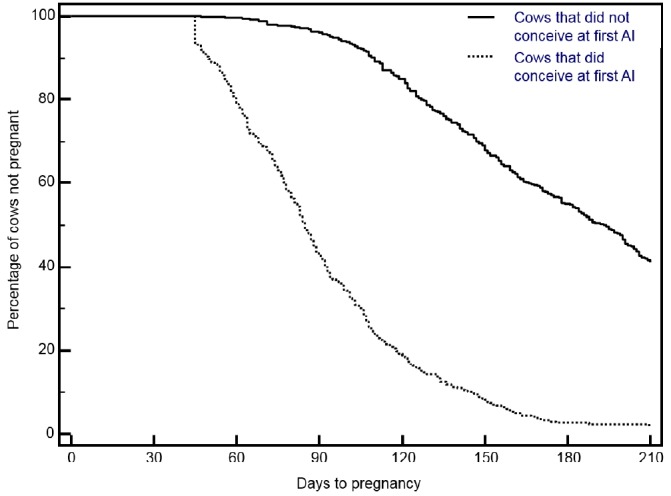
Survival curves for the interval between calving and conception in cows that did or did not conceive at their first AI. The probability of conception by 210 days postpartum was higher (hazard ratio: 21.81; 16.814 to 28.281; p<0.0001) in cows that did conceive than in those that did not conceive at their first AI. The median and mean days to conception were 193 and 172.6±2.0 in cows that did not conceive, and 85 and 91.8±2.0 in cows that did conceive at their first AI, respectively. AI, artificial insemination.

The survival curves showed that 41.8% of the cows were censored from the survival model because they were sold, died, or had not conceived by 210 days postpartum (Figure 1). The survival curves showed an 81 day extension in the mean calving to conception interval in cows that did not conceive (173 days) than in cows that did conceive (92 days) at their first AI. Cows that did conceive at first AI had a higher hazard ratio (HR = 21.81) than cows that did not conceive at first AI (p<0.0001) (Table 1). In addition, peri- and postpartum disorders and calving to first AI interval affected the hazard of conception by 210 days postpartum (p<0.0001) (Table 1).

### Risk factors limiting first service conception rate

Table 2 shows detailed statistics describing the factors influencing FSC in the two dairy farms. The logistic regression analysis revealed that lower BCS at first AI, higher temperature during the AI season, and the pre-existence of peri- or postpartum disorders were risk factors that limited FSC rate (Table 3). Cows with BCS <3.0 had a lower probability of conception at first AI (OR = 0.64, p<0.05) than cows with BCS≥3.0. Cows inseminated during summer were less likely to conceive (OR = 0.44, p<0.001) than cows inseminated during spring. Cows with peri- or postpartum disorders had a lower probability of conception (OR = 0.55, p<0.001) than cows without any of these disorders. However, farm, cow parity, BCS at 1 month postpartum, detection of a CL within 1 month of calving, year, timed AI, and calving to first AI interval were not associated with FSC rate (p>0.05, Table 3).

### Economic impact of first service conception

Table 4 shows the expense of reproductive treatment required until conception in cows that did or did not conceive at their first AI. Cows that failed to conceive required an extra $55.40 to be spent on reproductive treatment (using hormones including PGF_2a_, GnRH, and CIDR, semen, and palpation) than cows that did conceive at their first AI. Table 5 shows that an additional expense of $567.00 was incurred for other reproductive management procedures required to achieve conception (replacement heifers, nutrition, calf price, and labor) in cows that failed to conceive at their first AI. Thus, a total of $622.40 extra was spent on reproductive treatment and other management for cows that failed to conceive at their first AI.

## DISCUSSION

This study determined the risk factors limiting FSC rate and their economic impact in Korean dairy herds, which exhibit an increased milk yield under intensive production systems. Our data reveal that lower BCS, first AI taking place during summer, and the pre-existence of peri- or postpartum disorders are important risk factors limiting FSC rate. Moreover, a failure of FSC results in a mean loss of $622.40 per animal due to the additional expense incurred for reproductive treatment and other management (hormones, semen, replacement heifers, nutrition, and other costs).

The mean FSC rate (42.3%) in the present study was in the middle of the range of previously published rates (26.7% to 50.7%) [3,4]. Multiple logistic regression analysis revealed that lower BCS, and first AI during summer, and peri- or postpartum disorders limited FSC rate in our study. Cows with BCS<3.0 had a lower probability of conception at first AI than cows with BCS≥3.0, whereas BCS 1 month after calving did not affect FSC rate in the present study. Our results are consistent with some previous studies [3,18]. Cows with BCS≤2.25 were less likely to conceive at first service than cows with BCS ≥3.25 in a previous study [18]. In addition, the FSC rate was lower in cows with BCS of 1.5 to 2.0 than in cows with BCS of 3 to 4 in another [19], and FSC rate was higher in cows with BCS of 2.5 to 3.5 than in cows with BCS of 1.5 to 2.0 at the time of AI [3]. These findings indicate that nutritional status at the time of AI is very important for FSC and also imply that excessive BCS loss in early lactation should be recovered before performing AI. However, another previous study showed that BCS before calving affected the FSC: cows with a high BCS (≥3.5) had a higher FSC rate than cows with a low BCS (≤3.25) [8]. In addition, FSC rate was reported to be lower in cows with a BCS loss of >1.5 points than in cows with BCS loss of 1 to 1.5 points during the first month after calving, although no relationship between BCS at calving and FSC rate was shown [4]. The data from these two previous studies may suggest that the loss of BCS, reflecting a postpartum energy deficit, during early lactation could impair subsequent reproductive performance.

Our finding that cows inseminated during summer had a lower probability of FSC than those inseminated during spring is consistent with that of a previous study [20]. Moreover, several studies have demonstrated negative effects of heat stress on reproductive performance in dairy cows [21–23]. The greatest negative impact of heat stress on conception rate was observed during the 3-week period preceding insemination [22]. Another study reported that the effects of high temperature were greatest in the week immediately before and the one immediately after service [24]. Thus, the negative effects of heat stress on reproductive performance may be mediated through disturbed follicular development, inferior quality of the oocyte, a lower chance of fertilization, and/or embryonic or fetal loss [21,25].

Cows with peri- or postpartum disorders had a lower probability of conception at their first AI than cows without such disorders in the present study, consistent with previous findings [2,10]. One previous study showed that peripartum events, such as the birth of twins and stillbirth, reduced the probability of FSC [2]. Presumably, these peripartum events are associated with dystocia, which negatively affects the FSC rate [26]. Another study also demonstrated that peri- and postpartum disorders, such as retained placenta and metritis, and dystocia were associated with a reduction in FSC rate [10].

Herd, cow parity, resumption of cyclicity within 1 month of calving, year, timed AI, and calving to AI interval did not affect FSC in the present study. We observed that FSC rates were similar between the two farms studied, which is probably because they have similar nutritional and health management, and productivity. Our finding that cow parity does not affect FSC is consistent with those of some studies [4,19], but others have shown that FSC rate is affected by cow parity because it was lower in cows with parity ≥4 or 5 than in primiparous cows [7,10]. IDE or timed AI did not affect FSC rate in the present study, which is consistent with some previous studies [27,28]. However, other studies have shown that the FSC rate in cows undergoing AI during a detected estrus is higher than in cows undergoing timed AI following an Ovsynch protocol [17,29]. The reasons for these discrepancies among studies are not clear. However, they might relate to different herd management and practices, characteristics of animal, environmental conditions, such as weather or climate, or other factors. Contrary to our results regarding calving to first AI interval (<80 days vs ≥80 days), FSC rate was lower in cows with short calving to AI interval (<51 days and 51 to 95 days vs >95 days) in a Spanish study [10]. Similarly, another study showed that cows that underwent their first service before 60 days postpartum had a lower FSC rate than cows inseminated later [4]. These discrepancies might reflect the different categories of interval from calving to first AI used in each study.

We estimated the economic loss due to the failure of FSC by calculating the expense incurred by reproductive treatment and other reproductive management (hormones, semen, palpation, replacement heifers, nutrition, calf price, and labor), which was a total of $622.40 per animal in the present study. We found that a greater economic loss resulted from reproductive management measures (replacement heifers, nutrition, calf price, and labor) necessitated by the larger number of days open (81 days) than reproductive treatment (including hormones, semen, and palpation). A previous study showed that similar profitability was expected for cows that needed one or two inseminations per conception, but when more than three inseminations per conception were needed, the profit decreased by >$205/year per cow [6]. It is difficult to compare the economic loss between the previous study [14] and ours directly because of different study design and values for the animals and other products required for reproduction. However, it is clear that larger numbers of services per conception results in greater economic loss. In practice, the size of the economic loss may differ depending on the respective reproductive efficiency and the size of other expenses associated with reproductive management on dairy farms [30]. Nevertheless, our estimate of economic loss due to the failure of FSC should warn dairy managers and veterinary practitioners to recognize the importance of FSC and the necessity to adopt strategies to improve FSC in dairy herds.

In summary, our data show that lower BCS, hot weather at the time of first AI, and the pre-existence of peri- or postpartum disorders are important risk factors that limit FSC in Korean dairy herds, and that a failure of FSC is associated with a mean economic loss of $622.40 per animal. Thus, nutritional, environmental, and management strategies to maintain BCS ≥3.0, prevent heat stress during the insemination period, and reduce the incidence of or effectively treat peri- and postpartum disorders might be required to improve FSC rate in dairy herds with a high yield under intensive production systems, thereby reducing the cost of reproductive management.

## Figures and Tables

**Table 1:** Factors affecting the hazard of conception by 210 days postpartum analyzed by PHREG procedure

Variable	Hazard ratio	95% CI	p-value
BCS at 1 month postpartum			>0.05
Timed AI			>0.05
Peri- and postpartum disorders1)			
No	Reference		
Yes	0.67	0.548–0.811	<0.0001
Calving to first AI interval (days)			
<80	Reference		
≥80	0.15	0.118–0.191	<0.0001
Cows			
Did not conceive at first AI	Reference		
Did conceive at first AI	21.81	16.814–28.281	<0.0001

CI, confidence interval; BCS, body condition score; AI, artificial insemination.

1) Peri- and postpartum disorders include dystocia, retained placenta, septicemic metritis, clinical endometritis, ketosis, milk fever, and abomasal displacement.

**Table 2:** Details of the risk factors influencing first service conception rate in dairy cows

Variable	Level	No. of cows		

		AI	Conceived	%
Farm	A	350	157	44.9
	B	440	177	40.2
Cow parity	1	243	109	44.9
	2	206	85	41.3
	3	153	67	43.8
	≥4	188	73	38.8
BCS at 1 month postpartum	<2.75	126	47	37.3
	≥2.75	664	287	43.2
CL detection within 1 month postpartum	No	467	202	43.3
	Yes	323	132	40.9
Year	2011–2012	208	98	47.1
	2013–2014	294	119	40.5
	2015–2016	288	117	40.6
AI season	Spring	177	88	49.7
	Summer	156	45	28.8
	Autumn	255	105	41.2
	Winter	202	96	47.5
Timed AI	No1)	594	258	43.4
	Yes	196	76	38.8
Peri- and postpartum disorders2)	No	526	249	47.3
	Yes	264	85	32.2
Calving to first AI interval (days)	<80	347	143	41.2
	≥80	443	191	43.1
BCS at first AI	<3.0	183	62	33.9
	≥3.0	607	272	44.8

AI, artificial insemination; BCS, body condition score; CI, confidence interval.

1) Insemination at detected estrus (IDE).

2) Peri- and postpartum disorders include dystocia, retained placenta, septicemic metritis, clinical endometritis, ketosis, milk fever, and abomasal displacement.

**Table 3:** Odds ratios and variables included in the final multiple logistic regression model of the factors influencing first service conception rate in dairy cows

Variable	Odds ratio	95% CI	p-value
BCS at first AI			
≥3.0	Reference		
<3.0	0.64	0.449–0. 914	<0.05
AI season			<0.01
Spring	Reference		
Summer	0.44	0.279–0.702	<0.001
Autumn	0.73	0.493–1.079	>0.05
Winter	0.88	0.582–1.325	>0.05
Peri- and postpartum disorders1)			
No	Reference		
Yes	0.55	0.398–0.748	<0.001
Farm			>0.05
Cow parity			>0.05
BCS at 1 month postpartum			>0.05
CL detection within 1 month postpartum			>0.05
Year			>0.05
Timed AI			>0.05
Calving to first AI interval			>0.05

CI, confidence interval; BCS, body condition score; AI, artificial insemination.

1) Peri- and postpartum disorders include dystocia, retained placenta, septicemic metritis, clinical endometritis, ketosis, milk fever, and abomasal displacement.

**Table 4:** Costs of reproductive treatment required to achieve conception in cows that did or did not conceive at their first AI

Item	Unit	Value ($)/dose	Cows that did not conceive at first AI (n = 384)	Cows that did conceive at first AI (n = 334)
PGF_2a_	1 dose	3.5	1.98 doses×$3.5 = $6.93	1.39 doses×$3.5 = $4.87
GnRH	1 dose	2.5	2.14 doses×$2.5 = $5.35	1.49 doses×$2.5 = $3.73
CIDR	1 dose	22	0.49 doses×$22 = $10.78	0.14 doses×$22 = $3.08
Semen	1 straw	20	2.75 straws×$20 = $55.00	1 straw×$20 = $20.00
Palpation	1 time	7	4.36 palpations×$7 = $30.52	3.07 palpations×$7 = $21.50
Total			$108.58	$53.18

AI, artificial insemination; PGF_2a_, prostaglandin F_2a_; GnRH, gonadotrophin releasing hormone; CIDR, a controlled, internal drug-release device containing 1.9 g progesterone.

**Table 5:** Additional expenses for other reproductive management procedures in cows that failed to conceive at their first AI, incurred due to a larger number of days open

Item	Additional costs
Replacement	Difference between the value of cull cows ($1,500) and replacement heifers ($2,200): Cost of replacement per cow/d ($700.00×15.8%1)/425 days2)): $0.26 81 days×$0.26 = $21.06
Nutrition	Cost of nutrition per cow/d: $5.00 81 days×$5 = $405.00
Calf price	Calf price per cow/d ($100/425 days2)): $0.24 81 days×$0.24 = $19.44
Labor	Labor cost per cow/d: $1.50 81 days×$1.5 = $121.50
Total	$567.00

1) Culling due to infertility in cows that failed to conceive at first service: 72/456 (15.8%).

2) Calving interval in this study.
